# Supplementary material for: The interactome of intact mitochondria by cross-linking mass spectrometry provides evidence for coexisting respiratory supercomplexes
Source: Mol Cell Proteomics. 2017 Dec 8;17(2):216–32. doi: 10.1074/mcp.RA117.000470 (PMC5795388; doi:10.1074/mcp.RA117.000470)
Supplement: Supplemental Data [file supp_RA117.000470_133922_0_supp_23978_fzffwf.zip › spectra_annotation/mito_DR_spectra_annotation/338-1-18-1-5-1.pdf]

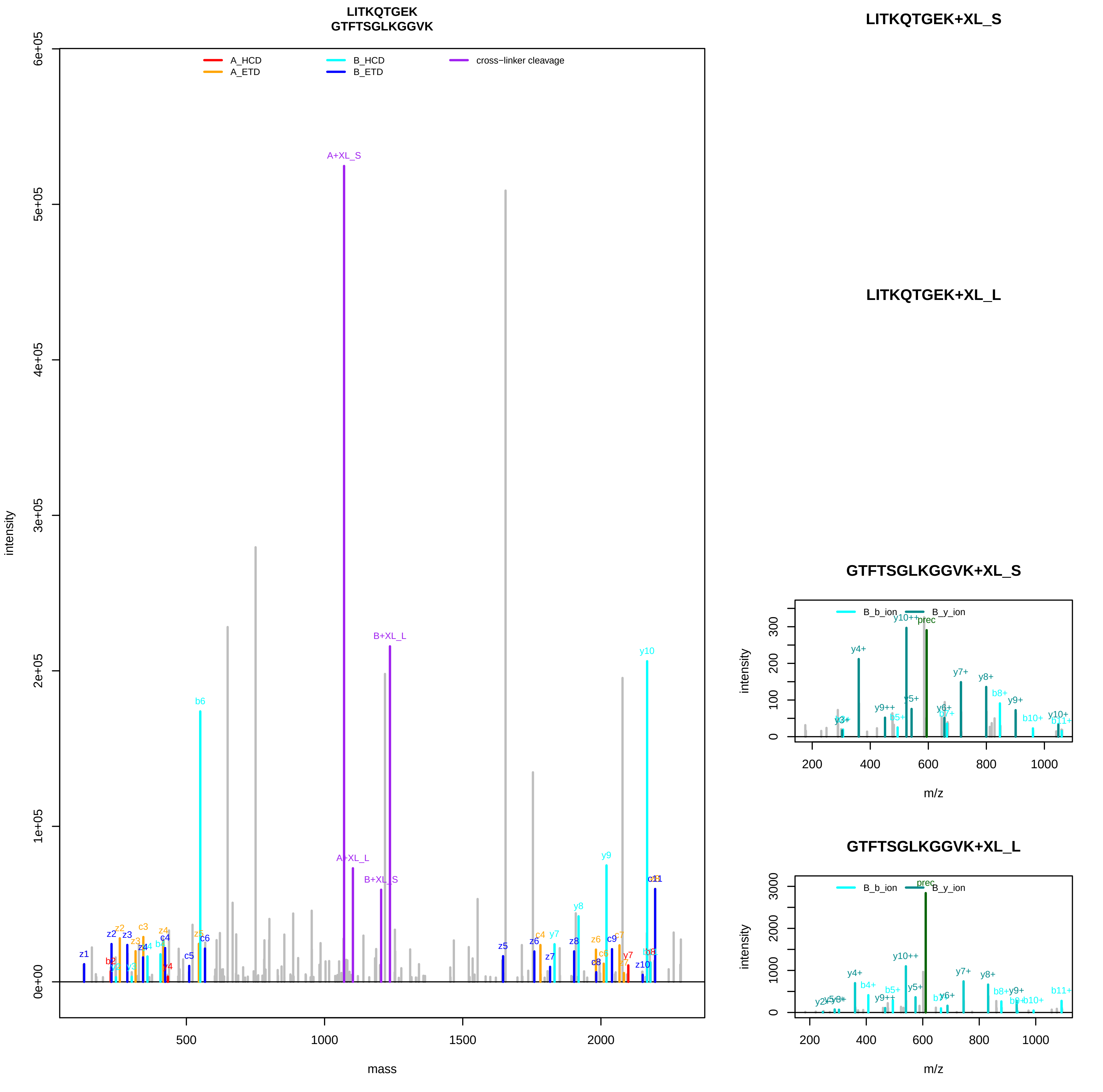

LITKQTGEK
GTFTSGLKGGVK
A_HCD
A_ETD
B_HCD
B_ETD
cross−linker cleavage
A+XL_S
B+XL_L
A+XL_L
B+XL_S
b6
y10
y9
y8
y7
c11
intensity
mass
LITKQTGEK+XL_S
LITKQTGEK+XL_L
GTFTSGLKGGVK+XL_S
B_b_ion
B_y_ion
prec
y10++
y4+
y7+
y8+
b8+
y9+
b10+
y10+
m/z
GTFTSGLKGGVK+XL_L
y10++
y4+
b4+
y7+
y8+
b11+
